# The use of residual serum samples to perform serological surveillance of severe acute respiratory syndrome coronavirus 2 in Dili and regional areas of Timor-Leste

**DOI:** 10.1093/trstmh/trac117

**Published:** 2022-12-09

**Authors:** Nevio Sarmento, Lourenço C Ico, Sarah L Sheridan, Maria Y Tanesi, Celia G Santos, Ismael Barreto, Nelia Gomes, Tessa Oakley, Anthony D K Draper, Nicholas S S Fancourt, Jennifer Yan, Kristine Macartney, Joshua R Francis, Paul Arkell

**Affiliations:** Global and Tropical Health Division, Menzies School of Health Research, Charles Darwin University, Darwin, NT 0810, Australia; Global and Tropical Health Division, Menzies School of Health Research, Charles Darwin University, Darwin, NT 0810, Australia; National Centre for Immunisation Research and Surveillance, Westmead, NSW 2145, Australia; Global and Tropical Health Division, Menzies School of Health Research, Charles Darwin University, Darwin, NT 0810, Australia; Department of Internal Medicine, Hospital Nacional Guido Valadares, Dili, Timor-Leste; Global and Tropical Health Division, Menzies School of Health Research, Charles Darwin University, Darwin, NT 0810, Australia; Global and Tropical Health Division, Menzies School of Health Research, Charles Darwin University, Darwin, NT 0810, Australia; Global and Tropical Health Division, Menzies School of Health Research, Charles Darwin University, Darwin, NT 0810, Australia; Global and Tropical Health Division, Menzies School of Health Research, Charles Darwin University, Darwin, NT 0810, Australia; Global and Tropical Health Division, Menzies School of Health Research, Charles Darwin University, Darwin, NT 0810, Australia; Global and Tropical Health Division, Menzies School of Health Research, Charles Darwin University, Darwin, NT 0810, Australia; National Centre for Immunisation Research and Surveillance, Westmead, NSW 2145, Australia; Global and Tropical Health Division, Menzies School of Health Research, Charles Darwin University, Darwin, NT 0810, Australia; Global and Tropical Health Division, Menzies School of Health Research, Charles Darwin University, Darwin, NT 0810, Australia

**Keywords:** COVID-19, remote areas, residual serum, SARS-CoV-2, serological surveillance

## Abstract

**Background:**

Lack of access to diagnostic testing for severe acute respiratory syndrome coronavirus 2 (SARS-CoV-2) infection can limit disease surveillance in remote areas. Serological surveillance can indicate the true extent and distribution of infections in such settings.

**Methods:**

This study monitored SARS-CoV-2 seroprevalence in residual serum samples salvaged from laboratories at five healthcare facilities across Timor-Leste from March to October 2021.

**Results:**

Seroprevalence increased from 8.3% to 87.0% during the study period. Potential immunity gaps were identified among children aged 0–15 y (who had not been eligible for vaccination) and individuals aged >60 y.

**Conclusions:**

Efforts to vaccinate vulnerable individuals including older people should be maintained. Residual serum samples can be analysed to give local, contemporary information about the extent and distribution of antibodies to infections, especially SARS-CoV-2, in areas where epidemiological information is limited.

## Introduction

From December 2019 to March 2021, community transmission of severe acute respiratory syndrome coronavirus 2 (SARS-CoV-2) was avoided in Timor-Leste through implementation of strict international border controls and quarantine measures. During this period, the capacity of the National Health Laboratory (NHL) in the capital, Dili, to conduct nucleic acid amplification tests was strengthened,^1,2^ where >200 000 tests were performed until February 2022. However, the rate of testing outside of Dili was low, resulting in a risk of undetected transmission in other regions. The AstraZeneca (ChAdOx1 nCoV-19, Belgium) viral-vector coronavirus disease 2019 (COVID-19) vaccine became available in April 2021, with the first doses administered to the majority of adults aged ≥18 y in Timor-Leste during the following 3 mo. Sinovac—Coronavac (China) inactivated COVID-19 vaccine became available in June 2021 but made up only a small minority (6%) of vaccine doses given in Timor-Leste (data as reported on 16 November 2021).^3^

## Materials and Methods

This study aimed to monitor the seroprevalence of SARS-CoV-2 across five referral healthcare facilities in Timor-Leste, including Dili and the regional centres of Baucau, Maliana, Suai and Oecusse. Residual serum samples that had been taken for other diagnostic purposes during March–October 2021 were deidentified and sent to the NHL where they underwent testing for SARS-CoV-2 anti-spike (anti-S) IgG using the qualitative Ortho Clinic Diagnostics chemiluminescent assay on the Vitros ECiQ platform (indicating either previous infection or any vaccination). Those that were anti-S positive were also tested using the qualitative Epitope Diagnostics Incorporated Novel Coronavirus COVID-19 ELISA to detect anti-nucleocapsid (anti-N) IgG antibodies (indicating previous infection or vaccination with a whole virus vaccine). Demographic data were collected from referral centres from the original pathology request form. We report the proportions of samples exhibiting seropositivity with 95% CIs.

## Results

**Figure 1.**
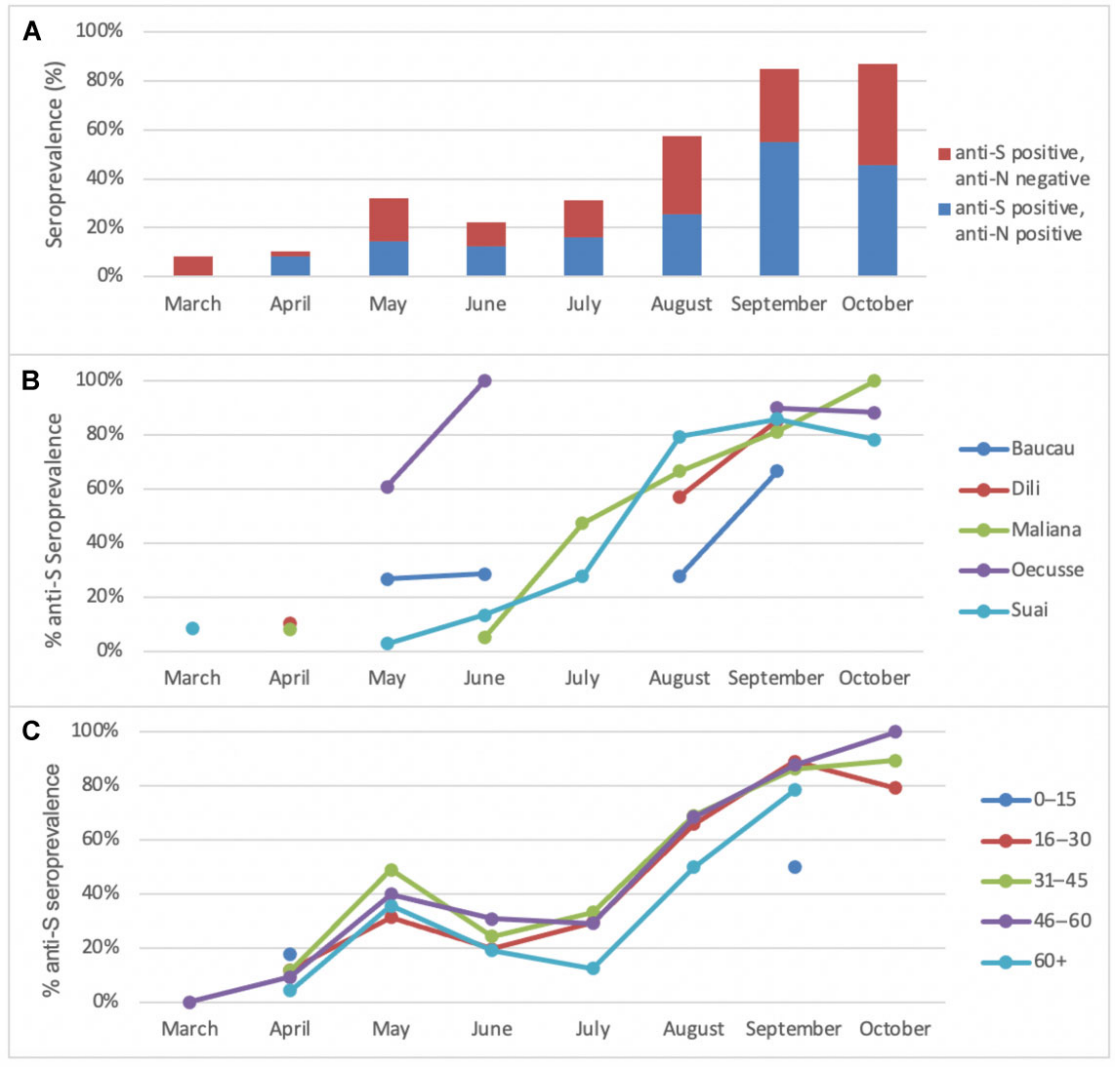
SARS-CoV-2 seropositivity from March to October 2021 among (A) the whole cohort (showing anti-S and anti-N seropositivity), (B) individuals presenting to different referral healthcare facilities (anti-S) and (C) individuals of different age groups (anti-S).

In total, 1652 samples were included in the study. Anti-S seroprevalence increased from 8.3% (95% CI 1.5 to 35.4%) to 87.0% (95% CI 77.7 to 92.8%) and anti-N seroprevalence increased from 0% (95% CI 0.0 to 24.5%) to 45.5% (95% CI 34.8 to 56.3%) during the study period (Figure 1A). Seroprevalence rose rapidly in the municipality of Oecusse, located within the western half of the island of Timor, with anti-S seroprevalence reaching 60.9% (95% CI 51.6 to 69.5%) by May 2021 and 100% (95% CI 86.7 to 100%) by June 2021, coinciding with the first wave of community transmission of SARS-CoV-2 (Figure 1B). Populations with relatively low anti-S seroprevalence at the end of the study included children (aged 0–15 y) who were not eligible for vaccination at the time (seroprevalence 50%; 95% CI 23.4 to 74.6%) and individuals aged >65 y (seroprevalence 78.6%; 95% CI 64.3 to 89.0%) (Figure 1C).

## Discussion

This study demonstrates that residual serum samples can be analysed to give local, contemporary information about the extent and distribution of antibodies to SARS-Co-V-2 in areas where epidemiological information is limited. A limitation of this approach is its reliance on samples taken from individuals who are seeking healthcare; hence findings may not be representative of the general population. As of October 2021, SARS-CoV-2 seroprevalence among healthcare attendees in Timor-Leste had risen to a high level (87.0%), which is a result of both naturally acquired infection and vaccination. This is in line with a recent longitudinal study of healthcare workers in Dili municipality that found a high uptake of vaccination and high anti-S seroprevalence by September 2021.^4^ To date, this is the only report of SARS-CoV-2 seroprevalence outside Dili municipality in Timor-Leste. There is some variability by geographic location and age, and it appears that very high seroprevalence was reached earlier in Oecusse, compared with other municipalities included in this study. Earlier and more widespread transmission in Oecusse during Timor-Leste's first large wave may be related to its close proximity to Indonesia and the large case burden there.^5^ Targeted vaccination efforts towards vulnerable individuals, including older people, should be maintained.

## Data Availability

Data and unpublished material can be accessed by contacting the corresponding author.
